# Alternative approaches for clinical clerkship during the COVID-19 pandemic: online simulated clinical practice for inpatients and outpatients—*A mixed method*

**DOI:** 10.1186/s12909-021-02586-y

**Published:** 2021-03-08

**Authors:** Hajime Kasai, Kiyoshi Shikino, Go Saito, Tomoko Tsukamoto, Yukiko Takahashi, Ayaka Kuriyama, Kazuhisa Tanaka, Misaki Onodera, Hidetaka Yokoh, Koichiro Tatusmi, Ichiro Yoshino, Masatomi Ikusaka, Seiichiro Sakao, Shoichi Ito

**Affiliations:** 1 Health Professional Development Center, Chiba University Hospital, Chiba, Japan; 2 Department of Respirology, Graduate School of Medicine, Chiba University, Chiba, Japan; 3 Department of General Medicine, Graduate School of Medicine, Chiba University, Chiba, Japan; 4 Department of General Thoracic Surgery, Graduate School of Medicine, Chiba University, Chiba, Japan; 5 Department of Medical Education, Graduate School of Medicine, Chiba University, Chiba, Japan

**Keywords:** Clinical education, Curriculum development/evaluation, Problem-based learning, Qualitative research methods, Quantitative research methods

## Abstract

**Background:**

The COVID-19 pandemic has created a need for educational materials and methods that can replace clinical clerkships (CCs) for online simulated clinical practice (online-sCP). This study evaluates the impact of using simulated electronic health records (sEHR) for inpatients, and electronic problem-based learning (e-PBL) and online virtual medical interviews (online-VMI) for outpatients, for an online-sCP using a learning management system (LMS) and online meeting system facilitated by a supervising physician.

**Methods:**

The sEHR was reviewed by medical students and subsequently discussed with a supervising physician using an online meeting system. In the e-PBL, medical students reviewed the simulated patients and discussed on the LMS. For the online-VMI, a faculty member acted as an outpatient and a student acted as the doctor. Small groups of students discussed the clinical reasoning process using the online meeting system. A mixed-method design was implemented. Medical students self-assessed their clinical competence before and after the online-sCP. They answered questionnaires and participated in semi-structured focus group interviews (FGIs) regarding the advantages and disadvantages of the practice.

**Results:**

Forty-three students completed the online-sCP during May and June 2020. All students indicated significant improvement in all aspects of self-evaluation of clinical performance after the online-sCP. Students using sEHR reported significant improvement in writing daily medical records and medical summaries. Students using e-PBL and online-VMI reported significant improvement in medical interviews and counseling. Students also indicated CCs as more useful for learning associated with medical interviews, physical examinations, and humanistic qualities like professionalism than the online-sCP. Eight FGIs were conducted (*n* = 42). The advantages of online-sCP were segregated into five categories (learning environment, efficiency, accessibility, self-paced learning, and interactivity); meanwhile, the disadvantages of online-sCP were classified into seven categories (clinical practice experience, learning environment, interactivity, motivation, memory retention, accessibility, and extraneous cognitive load).

**Conclusions:**

Online-sCP with sEHR, e-PBL, and online-VMI could be useful in learning some of the clinical skills acquired through CC. These methods can be implemented with limited preparation and resources.

**Supplementary Information:**

The online version contains supplementary material available at 10.1186/s12909-021-02586-y.

## Background

From February 2020, COVID-19 spread rapidly throughout the world, and on March 11, 2020, the World Health Organization declared it a pandemic [1]. Among the many ways this pandemic has affected society is its impact on medical education [2, 3]. In Japan, the number of COVID-19 cases and deaths increased from February to March [4], and on February 28, 2020, the Government instructed various schools to temporarily close beginning March 2 [5]. Consequently, most medical schools stopped clinical clerkships (CCs). Our institution, Chiba University Hospital, also canceled CCs from March 2020. Subsequently, we implemented online education for clinical practice (online-sCP) as an alternative, beginning May 8, 2020.

During CC, medical students have an opportunity to improve their clinical skills by experiencing medical interviews, performing physical examinations, writing medical records, and so on, as members of a medical team. It is unclear to what extent competencies such as medical interview and physical examination skills can be acquired through online-sCP, as medical students cannot physically meet and interact with patients. Furthermore, there is a lack of adequate resources, including time and materials, to prepare content complementary to the conventional curriculum for CCs, with all departments struggling to adapt. The Respiratory unit and General Medicine department of our institution have created simulated electronic medical records (sEHR) via Microsoft Excel for ease of use based on actual inpatient cases and electronic problem-based learning (e-PBL), and online virtual medical interviews (online-VMI) based on outpatient cases. These are being implemented for online-sCP in conjunction with Chiba University’s learning management system (LMS), Moodle, and an online meeting platform, Zoom™, with the supervising physician.

## Methods

### Aim

This study evaluates the feasibility and effectiveness of this approach, aiming to identify the advantages and disadvantages of online-sCP from the medical students’ perspectives.

### Ethics approval

This study was approved by the Ethics Committee of Chiba University (Approval No. 3425). The study database was anonymized, and the study complied with the requirements of the Japanese Ministry of Health, Labour and Welfare.

### Setting

#### Pre-clerkship course and CCs

At Chiba University’s School of Medicine, each grade comprises approximately 120 students. Medical schools in Japan have a six-year curriculum, and CC constitutes the last two years [6]. CC at the university begins in November of the fourth year and ends in October of the sixth year; students rotate from one department to another every four weeks. Table 1 shows the competencies set for CC.

**Table 1:** Comparison of the competencies for CC and online simulated clinical practice

Competencies related to the clinical clerkship	Respiratory unit (sEHR)	General Medicine (e-PBL and online-VMI)
The learner will be able to		
1. Take a patient’s primary medical history accurately.	○	○
2. Perform physical examination of adults and children and basic clinical procedures properly.		
3. Diagnose disease based on clinical reasoning.	○	○
4. Select tests and interpret their results to diagnose and treat common diseases.	○	○
5. Develop an appropriate treatment plan for common diseases.	○	○
6. Write medical documents and present the clinical course of patients appropriately.	○	
7. Perform safe medical treatment using evidence-based medicine.	○	○
8. Participate in informed consent and patient education.		○
9. Participate in diagnosis, treatment, and systemic management.		

*e-PBL* electronic problem-based learning, online-VMI: online virtual medical interview, *sEHR* simulated electronic health records organized by Microsoft Excel

In March 2020, CC was canceled due to the pandemic. Following this, the university conducted online-sCP for fifth- and sixth-year medical students between May and July.

### Participants

Among 118 fifth-year medical students in 2020, only medical students who participated in the online-sCP for the Respiratory unit and General Medicine were included. A group of 10 or 11 medical students underwent a four-week training program as members of a medical team of doctors and residents until February 28, 2020. Between May and July 2020, a four-week online-sCP was conducted in the Respiratory unit and General Medicine departments. Hybrid CC at the hospital resumed after July 2020, consisting of online-sCP and in-person CCs. We implemented several faculty developments in advance for the supervising physicians and standardized the course content.

We explained how we would use the participants’ questionnaire responses through written and verbal instructions transmitted online, along with the questionnaire, to the students. Informed consent was then obtained along with the students’ responses.

### Online learning procedure

#### Department’s CCs and online-sCPs

Table 2 shows an overview of online-sCPs and CCs in each department. Over the course of four weeks, online-sCPs using sEHR were conducted in the Respiratory unit, and online-sCPs using e-PBL and online-VMI were conducted in General Medicine.

**Table 2:** Comparison of the weekly schedule for CC and online-sCP

	Respiratory Unit		General Medicine	
	CC	Online-sCP	CC	Online-sCP
**Duration**	4 weeks	4 weeks	4 weeks	4 weeks
**Number of Students**	7–8	11	10–11	10–11
**Number of** **Attending Physicians**	4 (Respiratory Medicine)	4 (Respiratory Medicine) + 1 (Thoracic surgery)	2	2
**Patient in charge**	2–3 in four weeks (Inpatient)	4 in 4 weeks	4–5 in a week (Outpatient)	1 in a week
**Weekly task**	(Everyday) Medical care and documentation of medical records for assigned case. (Once a week) Rounds with an attending physician.	(Everyday) Documentation of medical records for assigned case.	(At least 1 time /week) Mini-CEX	(Everyday) Discussion post
**Overall task**	2 Summaries of case, 1 Report	2 Summaries of case, 1 Report	Portfolio	Portfolio
**Schedule**	(Everyday) Lecter (60 min) (Once every week) Conference Hospital ward round (Once every 4 weeks) Outpatients practice Bronchoscopy	(Everyday) Practice using sEHR 8:30 sEHR upload in LMS ↓ 30–60 min Meeting with attending physician on Zoom™ ↓ ~ 17:00 Students upload their medical records to LMS Lecter (60 min)	(Everyday) Outpatient Practice Debriefing (Once every week) Problem-based Learning Clinical Reasoning Conference	(Everyday) e-PBL on LMS (Three times a week) online-VMI (Once every week) Clinical Reasoning Conference

*CC* clinical clerkship, *e-PBL* electronic problem-based learning, *LMS* learning management system, *online-sCP* online simulated clinical practice, online-VMI: online virtual medical interview, *sEHR* simulated electronic health records organized in Microsoft Excel

The online-sCPs for the Respiratory unit and General Medicine were conducted to provide an opportunity to learn the clinical activities shown in Table 1. The sEHR was especially targeted to ensure students learn to take a patient’s primary medical history accurately, write medical documents, and present the clinical course of patients appropriately. The e-PBL and online-VMI targeted learning to take a patient’s primary medical history accurately, diagnosing diseases based on clinical reasoning, and selecting tests and interpreting their results to diagnose and treat common diseases.

#### sEHR

**Fig. 1:**
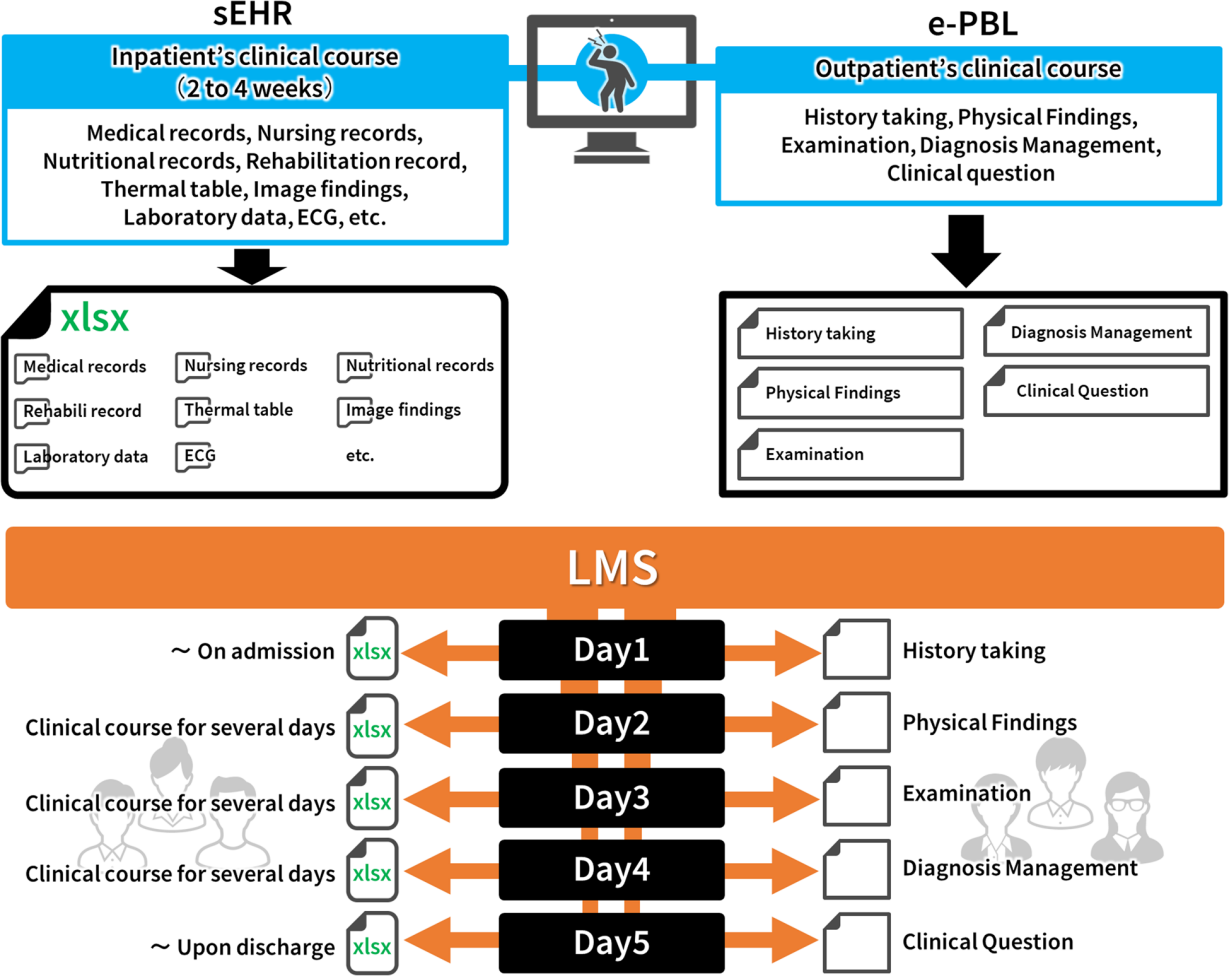
Process of making a simulated patient’s medical record and schedule in sEHR and e-PBL. e-PBL: electronic problem-based learning, LMS: learning management system, sEHR: simulated electronic medical records via Microsoft Excel for ease of use

Three cases each of respiratory medicine (i.e., complications associated with infectious pneumonia and acute exacerbation of chronic obstructive pulmonary disease, pulmonary thromboembolism, and chemotherapy for lung cancer) and thoracic surgery (i.e., lung cancer, mediastinal tumor, and pneumothorax) were selected as shown in Fig. 1. Information from medical professionals’ electronic health records (EHR), image findings, and examination results were added to the Excel worksheets, and any information that could identify individuals was excluded or changed to fictitious data. The clinical course for each case ranged from seven to fifty-five days and was divided into five segments based on each case. Each simulated case record was arranged to address the course’s learning purpose and the five-day practice.

#### sEHR programming

As reflected in Supplementary Fig. 1, the participants attended an orientation using an online meeting. In the Respiratory unit, one case per week was shared by two to three students, and one surgical case per week was assigned to each student. Every morning, an sEHR, according to the practice day, was uploaded to the LMS; the students reviewed the information and made an assessment based on the results of various examinations pertaining to the case. They also took into consideration medical interviews and physical examinations. Online meetings were subsequently held with group members and the supervising physician. After sharing the results for the tasks assigned the previous day, the meeting continued as follows: 1) the student summarized the clinical course of the case; 2) the student was asked to list the necessary questions and examinations required, and the supervising physician answered with the findings for those items; 3) an analysis was conducted of the examinations performed; and 4) the student discussed the treatment plan. To conclude, students were asked to decide the charge for tasks related to the case. Each online meeting ranged from 30 to 60 min. The students created a medical record based on the sEHR and the meeting and uploaded the record onto the LMS by evening. The supervising physician added feedback on the submitted records. The meetings and feedback were supervised by four of the authors of the study (HK, YT, GS, AK).

#### E-PBL

The e-PBL was based on outpatient cases that involve biological, psychological, and social issues and was conducted through the LMS (Fig. 1, Supplementary Fig. 2). The e-PBL is another variation of the standard-style PBL. Twenty outpatient cases were selected from actual cases experienced in General Medicine. One case per week was shared by two or three students. Additional medical history, physical examination findings, and laboratory findings were shared with students daily. Then, based on peer-assisted learning pedagogy, the students discussed the tasks in small groups [7, 8].

Each group reviewed the case, key clinical information that formed the basis of the differential diagnosis, and additional clinical information to identify potential diseases. They posted weekly assignments on a discussion board on the LMS, in which students were asked to formulate clinical questions and post evidence for them. Participants were encouraged to view cases from other groups on the LMS and actively participate in discussions.

#### Online-VMIs

Online-VMIs are an impromptu style of patient simulation encounters. To ensure that the appropriate history was collated, and physical examinations were conducted as needed, it was necessary to practice conducting medical interviews. In these sessions, a faculty member acted as a patient and a student played the role of a doctor during a medical interview that took the form of an online meeting (Supplementary Fig. 3). To achieve cost efficiency in simulated medical interviews, it is common for standardized patients to play the role of the patient. However, we adopted the impromptu style because of the prevalence of COVID-19, which made it difficult to recruit sufficient numbers of patients, and faculty members played the role of patients. The supervising physician provided clinical information to the medical student. During the physical examination, the medical students asked the supervising physician for information about focused physical findings based on their clinical hypothesis. At the students’ request, the supervising physician presented them with the physical findings of the simulated patients, with photographs and videos (e.g., inspection of the pharynx, nystagmus, and deep tendon reflexes, etc.). A number of students from the same group could observe the interview, thereby promoting peer-assisted learning. By using the chat function in the video conference system, students could share the clinical reasoning process. Furthermore, they were able to have small group discussions regarding the clinical reasoning process by using a breakout room. This activity was conducted three times a week for an hour per session. Twelve cases were used that involved common diseases encountered in primary care settings.

### Data collection

#### Quantitative data collection

To evaluate online-sCP’s effect on students, quantitative data were compiled using a questionnaire. Before and at the end of the online-sCP for each department, participants completed a questionnaire (Table 3). Clinical skills competence constituted nine items, including seven items related to the mini-clinical evaluation exercise (Mini-CEX) and the ability to write medical records and a summary of the case.

**Table 3:** Questionnaire items at the beginning of and after the online-sCP

Question	Responses
**At the beginning of the online-sCP**	
(A1) What is your self-assessment of your clinical skills competence? Medical interviewing, physical examination, humanistic qualities/professionalism, clinical judgment, counseling, organization or efficiency, overall clinical competence, writing daily medical records, writing medical summaries	Nine-point Likert scale 1 (extremely poor) to 9 (extremely good)
(A2) What is the duration of self-study per day, other than the time spent on practical training during CC thus far?	The response options are as follows: ≤1 hour, 1–2 hours, 2–3 hours, 3–4 hours, 4–5 hours, 5–6 hours, ≥6 hours
(A3) How long was the average duration of self-study time per day during the period of CC postponed	The response options are the same as in question A2.
**At the end of the online-sCP**	
(B1) What is your assessment of your clinical skills competence? Same items as in question A1.	Nine-point Likert scale 1 (extremely poor) to 9 (extremely good)
(B2) What is your level of satisfaction with the practice? Entire program of the department For Respiratory unit: sEHR For General Medicine: e-PBL, online-VMI	Five-point Likert scale 1 (strongly disagree) to 5 (strongly agree)
(B3) What was the average duration of self-study time per day during the online-CP?	The response options are same as in question A2.
(B4) Which of these do you think is more useful for CC or the online-CP? Same items as in question A1.	Five-point Likert scale 1 (CC) to 5 (online-sCP)

*CC* clinical clerkship, *e-PBL* electronic problem-based learning, *LMS* learning management system, *online-sCP* online simulated clinical practice, *online-VMI* online virtual medical interview, *sEHR* simulated electronic health records organized in Microsoft Excel

#### Qualitative data collection

To evaluate the strengths and weaknesses of online-sCP, we performed focus group interviews (FGIs) with medical students. At the end of the online-sCP, the students were interviewed during semi-structured focus groups regarding the advantages and disadvantages of the program, with this qualitative phase used to help explain quantitative data results. The participants comprised eight groups of medical students (43 whole student cohorts in total). The criteria for participant selection specified that all medical students were to be included, as the target population had to be homogeneous in order to investigate perceptions regarding online-sCP. Students were recruited to participate in focus group sessions after the online-sCP ended.

FGIs were conducted by two physician-researchers (HK and KS) and recorded independently using an iteratively created interview guide. Students were asked the following questions: *1) “Think of the advantages of online-sCP and why do you believe that these are advantages?”; 2) “Think of the disadvantages of online-sCP and why do you believe that these are disadvantages?”* (Supplementary Table 1). The interview guide was validated by two researchers (HK and KS) prior to data collection.

The interviews took no longer than 30 min and considered work impact and participant fatigue. A Zoom™ video recording system was used to record the interview with participant permission. The interviews were transcribed verbatim.

### Data analysis

#### Statistical analysis

Quantitative data was expressed in terms of the mean ± standard deviation (SD), unless otherwise indicated. The Wilcoxon signed-rank test was used to compare parameters before and after the online course. Pearson’s chi-square test was used to compare self-study time during the online-sCP, CC, and period during which the CC was postponed. A *p*-value < 0.05 was used. All statistical analyses were performed using JMP 13.0 (Cary, North Carolina, USA).

#### Qualitative content analysis

In line with previous studies, qualitative content analysis was used to analyze FGI transcripts [9]. This analysis comprises descriptions of the manifest content and interpretations of the latent content [10]. HK and KS independently read and coded all transcripts. Subsequently, they discussed, identified, and agreed on the coding of the descriptors. Interrater reliability was measured using the Kappa coefficient [11] [0.8–1.0 = almost perfect; 0.6–0.8 = substantial; 0.4–0.6 = moderate; 0.2–0.4 = fair].

## Results

A total of 43 medical students conducted online-sCPs for the Respiratory unit and General Medicine in May and June 2020 (22 and 21 students, respectively). All 43 students completed the questionnaire. While one of the medical students was unable to participate in the online FGI due to an unintentional network communication failure, eight semi-structured FGIs were conducted (*n* = 42) (Supplementary Fig. 4).

### Questionnaire results

**Fig. 2:**
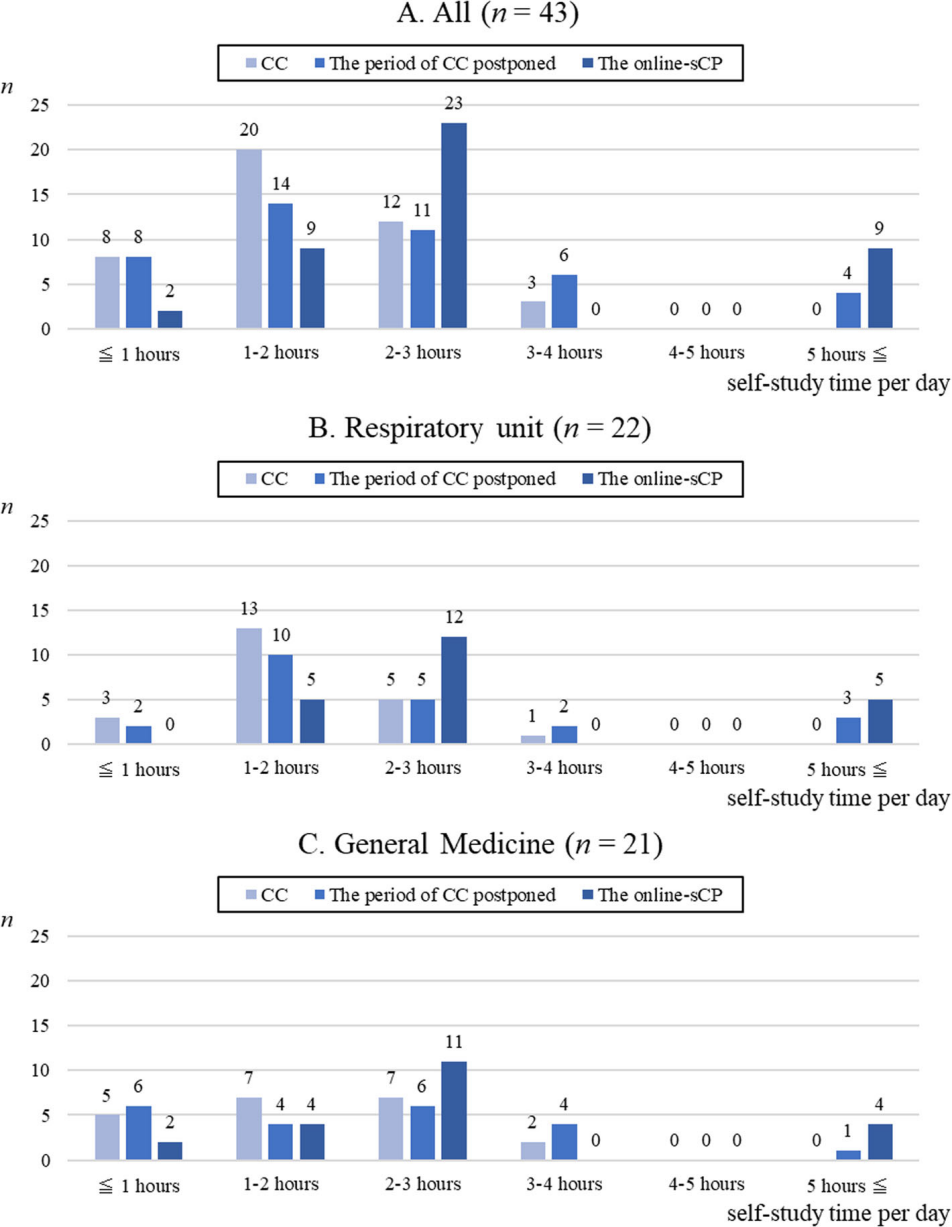
Self-study time per day during CC, CC discontinuation, and online-sCP. CC: clinical clerkship, online-sCP: online clinical simulated practice

Questionnaire responses indicate that the students’ satisfaction level with the online-sCP conducted by each department was acceptable (all, 4.8 ± 0.5; Respiratory unit, 4.9 ± 0.3; General Medicine 4.7 ± 0.6). The satisfaction level with sEHR, e-PBL, and online-VMI was also acceptable (4.5 ± 0.5, 4.8 ± 0.4 and 4.8 ± 0.4, respectively). Self-study time, other than the time spent participating in practice per day, tended to be longer during online-sCP than during standard CC or the period when CC was postponed (*p* = 0.140 and *p* = 0.114, respectively; Fig. 2)

**Fig. 3:**
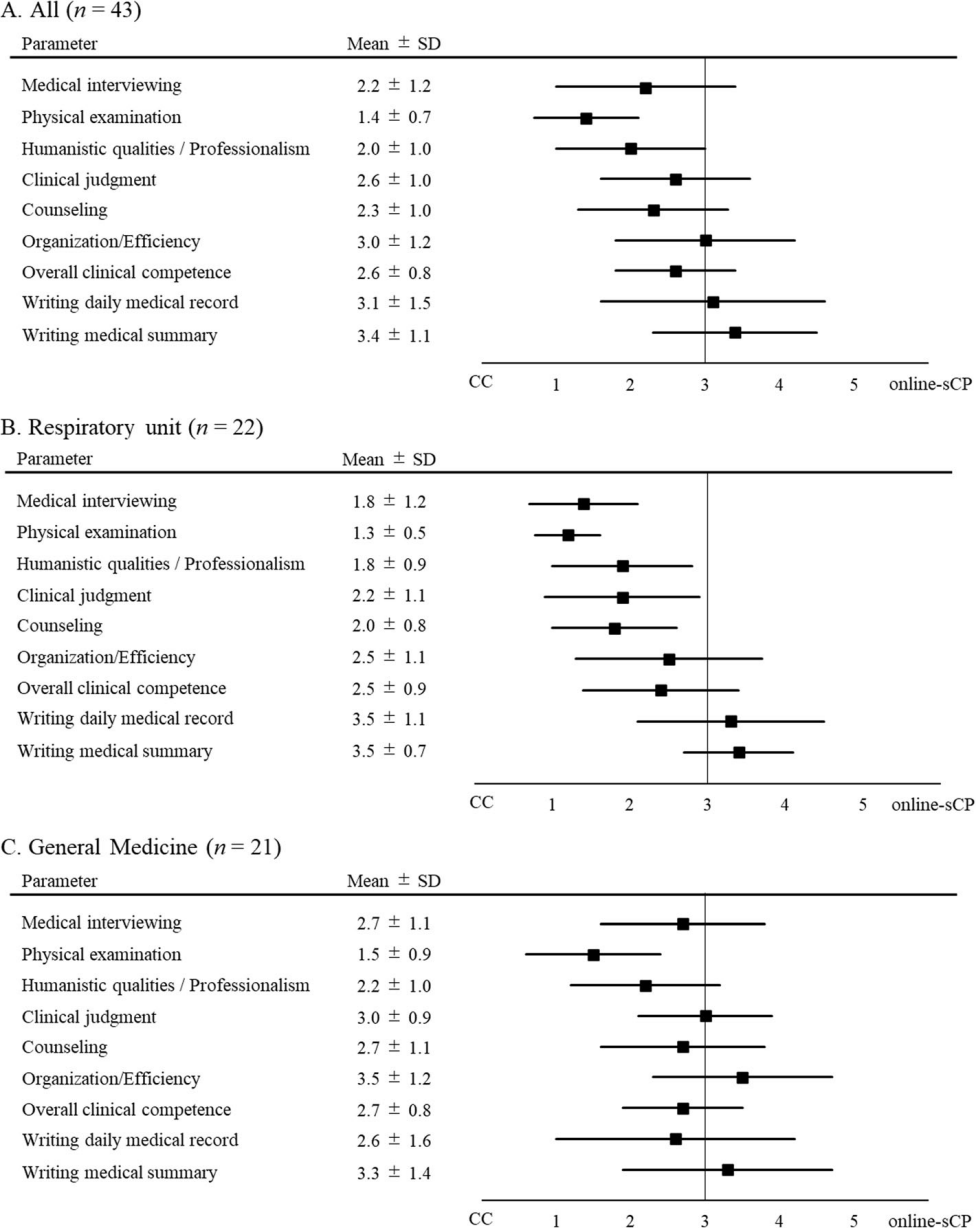
Comparison of CC and online-sCP to improve clinical competence assessed by medical students. CC: clinical clerkship, online-sCP: online clinical simulated practice, SD: standard deviation

Students indicated the CCs as more useful for learning associated with medical interviews, physical examinations, and humanistic qualities like professionalism than the online-sCP. They stated that the online-sCP was more useful for learning based on the approaches. Based on the scoring, sEHR was preferred for writing daily medical records and medical summaries. The e-PBL and online-VMI were scored higher by students in terms of learning organization and efficiency, and for writing medical summaries, than CC (Fig. 3).

**Fig. 4:**
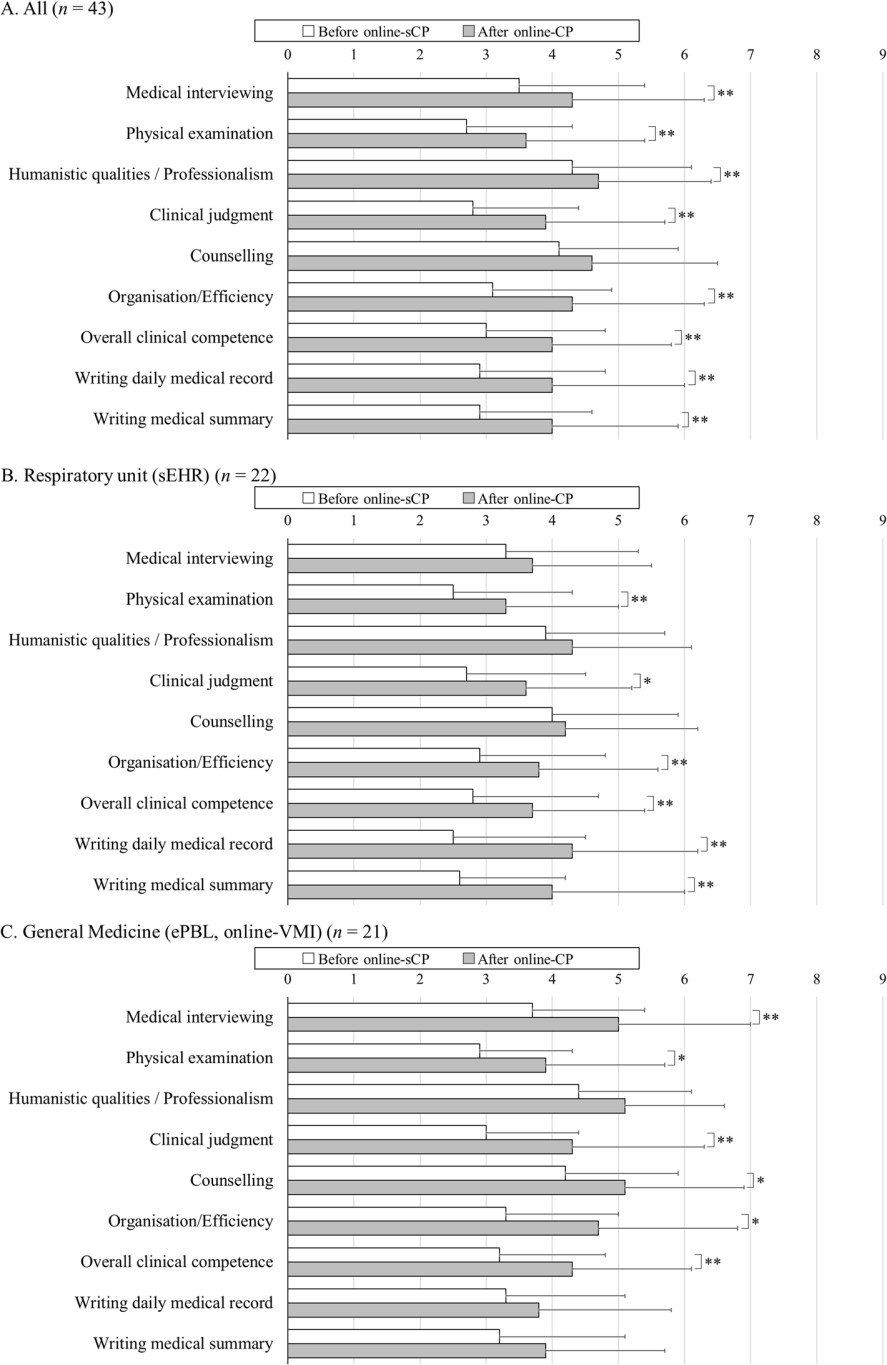
Changes in clinical performances according to the medical students’ self-assessment. Data are presented as mean ± standard deviation. * *p* < 0.05, ** *p* < 0.01. e-PBL: electronic problem-based learning, LMS: learning management system, online-sCP: online clinical simulated practice, online-VMI: online virtual medical interview, sEHR: simulated electronic health records organized in Microsoft Excel

Figure 4 summarizes the self-evaluation of participants’ medical performance before and after the online-sCP. All students indicated significant improvement for all aspects of clinical performance after the online-sCP. In the Respiratory unit, students indicated improvement in writing daily medical records, and their medical summaries improved significantly (from 2.5 ± 2.0 to 4.3 ± 1.9, *p* < 0.001; from 2.6 ± 1.6 to 4.0 ± 2.0, *p* < 0.001, respectively). In General Medicine, students indicated improvement in medical interviews, and counseling improved significantly (from 3.7 ± 1.7 to 5.0 ± 2.0, *p* = 0.009; from 4.2 ± 1.7 to 5.1 ± 1.8, *p* = 0.043, respectively).

### FGI results

The advantages of online-sCP were segregated into five categories (learning environment, efficiency, accessibility, self-paced learning, and interactivity) and twenty concepts (Table 4). Seven categories (clinical practice experience, learning environment, interactivity, motivation, memory retention, accessibility, and extraneous cognitive load) and 31 concepts associated with the disadvantages of online-sCP were analyzed (Table 4). The results of the interrater reliability were Kappa = 0.92. Three common categories (learning environment, interactivity, and accessibility) were observed in the two analyses, reflecting the advantages and disadvantages of online-sCP. The advantages included self-paced learning and efficiency, while the disadvantages were extraneous cognitive load and lack of clinical practice experience.

**Table 4:** Advantages and disadvantages of online simulated clinical practice (*n* = 42)

Categories	Concepts	Frequency	Total
**Advantage**			
Learning environment	Establishing self-learning rhythm	10	35
	Awareness from the perspective of peers	9	
	Psychological safety in learning space	4	
	Sufficient problem-solving time	4	
	Frequent feedback	3	
	Sufficient discussion time	3	
	Timely feedback	1	
	Sufficient contact time with attending physicians	1	
Efficiency	Well-structured study case	8	26
	Summarized clinical course of study case	7	
	Reduced commute time to school	6	
	Effective use of free time	5	
Accessibility	Timely access to learning resources	13	22
	Comfortable contact with attending physicians	7	
	Accessibility to electronic medical records	2	
Self-paced learning	Learning at learner’s own pace	7	17
	Arrangement for learning time	6	
	Sufficient write time for medical records	4	
Interactivity	Communication tool	5	10
	Peer engagement	5	
**Disadvantage**			
Clinical practice experience	Difficulty in observation of physician’s practice	9	48
	Difficulty in feeling the atmosphere in the clinical setting	8	
	Hard to perform physical examination	7	
	Lack of patient communication	6	
	Lack of clinical experience in real settings	5	
	Lack of medical interviews	4	
	Lack of clinical procedures	3	
	Lack of patient complexity	3	
	Lack of presentation	2	
	Inexperience with professionalism	1	
Learning environment	Network disturbance	5	15
	Low video quality	3	
	Difficulty in speaking up	3	
	Poor audio quality	3	
	Without timely feedback	1	
Interactivity	Not being able to attend clinical conferences	4	9
	Lack of interaction between students	2	
	One-way lecture	2	
	Limit on non-verbal communication	1	
Motivation	Lack of tension	6	9
	Lack of clinical experience	2	
	Lack of reality	1	
Memory retention	Hard to imagine real patient	3	8
	Not being connected to the clinical experience	2	
	Difficulty in imagining procedures	2	
	Connection to clinical experiences	1	
Accessibility	Difficulty in asking questions	4	6
	Distance between student and attending physician	1	
	Difficulty in finding learning resource	1	
Extraneous cognitive load	Unfamiliarity with online learning	3	6
	Difficulty in filling out medical records	2	
	Time to get used to tool	1	

## Discussion

This study evaluated the feasibility and effectiveness of online-sCP as an alternative to CC for outpatient and inpatient care, and identified the advantages and disadvantages of online-sCP for medical students. Its two main findings are as follows. First, online-sCP with sEHR, e-PBL, and online-VMI are not only effective in terms of the clinical performance acquired through CC that was reported in the students’ self-assessments but also an efficient learning method for medical students to productively use their time. Second, although online-sCP is not a satisfactory alternative for CC, it can provide a clinical learning experience for students.

Online-sCP using sEHR for inpatient care can be a useful method to learn how to write medical records and summaries for students and is a resource-effective method. In CCs, medical students must use EHRs to evaluate and organize varied information such as medical records and the results of various examinations. They also have to holistically assess their patients. EHR systems are a commonly used health record format [12], and proper documentation of health records is an important skill [12, 13]. In this study, students claimed improvement in writing daily medical records and medical summaries in self-assessments, and this result was consistent with the main goal of the sEHR, which is improving writing medical records. Furthermore, by shortening the clinical course to five days to fit the duration of the practice period, even in cases of prolonged hospitalization lasting more than a few weeks, the supervising physician could efficiently focus on what students should learn from the case.

Microsoft Excel was used as the format for sEHRs, as varied medical information can be assigned to different worksheets, similar to the interface of an actual EHR. Such spreadsheet software is a common application that can be widely used in any environment. Therefore, the sEHR can be created without any additional resources if the EHR data of the actual cases are available.

Online-sCP in the form of e-PBL and online-VMI providing simulated outpatients could be an effective and efficient educational method to garner an understanding of clinical reasoning. Through e-PBL and online-VMI, performance regarding medical interviews, clinical judgment, and organization or efficiency in self-assessments of the students improved significantly. The primary objective is to improve students’ clinical skills to take a patient’s primary medical history accurately, diagnose disease based on clinical reasoning, and select tests and interpret their results to diagnose and treat common diseases. Medical students must seek solutions based on the clinical information presented to them daily, sort the problems, and thereby, identify solutions. In this study, this process allowed an opportunity for students to learn about organization or efficiency and clinical judgment, even in online-sCP. While e-PBL is a paper-based and problem-solving skills training, online-VMI contributes to learning skills during patient interactions. Online meeting systems may contribute to the improvement of skills required for medical interviews and clinical reasoning, even in situations where actual face-to-face interaction and education are difficult to provide. In both the educational methodologies, peer-assisted learning has been used [14]. In particular, peer-assisted learning has been applied to problem-based learning [7, 8]. Peer-assisted learning supports student learners’ cognitive, psychomotor, and affective development, which leads to an improvement in clinical reasoning skills [15].

An important thing to note is that the present study was a single-arm study, and no group of non-participating students was compared with the participating group. Additionally, the clinical competencies of medical students were evaluated only through self-evaluation; the students were not evaluated on their actual clinical performance. Although this assessment is subjective, there are currently no established criteria to evaluate clinical competencies in online settings. This is due to the lack of established criteria for assessing performance online and the fact that the online-sCP was initiated without the adequate preparation of an assessment method. Ideally, a comparison of clinical performance between online-sCPs and standard CCs would be desirable; however, this was difficult for the above reasons. Future studies should evaluate the synergistic effect of online-sCP on CC by comparing CC combined with online-sCP against CC alone.

This study presented online-sCP as an efficient learning method for medical students to productively use their time through FGIs. The advantages of sEHR, e-PBL, and online-VMI were predicted to be that the learning goals could be established easily by selecting patients for better learning and adding or modifying the clinical course of the case. In fact, during FGIs, students identified the online-sCP as a well-structured learning method that effectively summarized the clinical course. Additionally, in the online-sCP, medical students even had the opportunity to take the time to assess serious and urgent cases. During actual CC, depending on the urgency of the patient and the number of EHR devices, medical students do not always have sufficient time to assess the patient and create medical records. Furthermore, medical students did not have to spend time commuting to the hospital and waiting to attend events such as conferences and examinations. Consequently, they had more time to study. During the FGI, the students also stated that accessibility and self-paced learning were advantages of online-sCP. Furthermore, as a number of students reviewed the same case and participated in the discussion, each student’s assessment could be shared, and proper peer review could be carried out. Although our methods have a large element of educational material that defines the content, these methods have the potential to be customized as needed in order to ensure uniformity of opportunity.

In online-sCP, the lack of clinical practice opportunities that involve actual patients is a matter of concern. This makes it difficult to train students in tasks such as taking medical history, performing physical examinations, and explaining results to patients. In this study, the lack of opportunities to practice physical examination is a limitation. Medical students stated that CC provides proper opportunities for the same, unlike online-sCP. The students stated in the FGI that the lack of clinical practice experience is a disadvantage of online-sCP. However, in e-PBL and online-VMI, medical students had the opportunity to analyze the necessary questionnaires and physical examinations of patients before the online meeting with the supervising physician, and the supervising physician responded to the results of these questionnaires and physical examinations. Furthermore, in the case of online-VMI, students can take the patient’s history and explain the results “through the screen.” Additionally, medical students can review their own medical interviews, and peer reviews can be performed by watching the medical interviews of other students. Through the chat function, students can also support each other and discuss the process of clinical reasoning. Possibly based on these advantages, a larger number of students from General Medicine than from the Respiratory unit responded that online-sCP was a more effective method of learning medical interviewing, clinical judgment, counseling, and organization/efficiency skills than CC. However, one of the disadvantages stated was students’ lower memory retention in terms of connecting the clinical experience and real patients, as they could not interact with patients in FGI. Yet, accessibility and retention of information in the online environment could be improved by technical advancement. Although it is not possible for online-sCP to completely replace CCs, it may be possible to leverage the learning effectiveness of CC in online-sCP with some ingenuity.

It is important to note is that the two approaches implemented in this study as alternatives to CC have the potential to be used as supplements with CC and as a teaching tool before CC once the COVID-19 pandemic is over. Since it is unclear when the situation will be under control and there is a possibility of a recurrence of the pandemic due to various factors, such as intensive care capacity, development of vaccines and treatments, and the influx of visitors from foreign countries [16, 17], CC may be discontinued again. Furthermore, some medical students may not want to return to the clinical setting under the current circumstances [18]. Therefore, the demand for online alternatives will be high. This situation could be taken as a learning opportunity to review conventional “clinical practice” and introduce a more effective and efficient methodology. This could be the first step toward innovation in the post-COVID-19 era.

This study has three main limitations. First, in terms of research design, this was a single-site study in an uncontrolled environment and relied partly on students’ self-assessment for data collection. Second, since it was only conducted at a single university in Japan, the responses are subject to cultural bias and the participants’ learning environments. Third, the self-study time during CC and the period when CC was postponed were drawn from the students’ memories, because there were no subjective data, such as those kept in a portfolio. Students had been attending CCs for 2–3 months before the online-sCP, and their responses regarding their self-study time during CCs can be ambiguous.

## Conclusions

Online-sCP with sEHR, e-PBL, and online-VMI could be useful in learning some of the clinical skills acquired through CC. These methods can be implemented with limited preparation and resources. Furthermore, these methods have the potential to serve as efficient and sustainable educational methods that can be used in extreme situations.

## Supplementary Information

**Additional file 1 **: **Supplementary Fig. 1** Daily flow of online clinical practice using sEHR. LMS: learning management system, sEHR: simulated electronic health records organized in Microsoft Excel. **Supplementary Fig. 2** Electronic problem-based learning using the LMS. LMS: learning management system. **Supplementary Fig. 3** Process of online virtual medical interviews. **Supplementary Fig. 4** Flow diagram for the study. Online-sCP: online simulated clinical practice.

**Additional file 2.**

## Data Availability

Data sharing is not applicable to this article as no datasets were generated or analyzed during the current study.

## Abbreviations

CC: Clinical clerkship
e-PBL: Electronic problem-based learning
FGI: Focus group interview
LMS: Learning management system
Mini-CEX: Mini-clinical evaluation exercise
online-sCP: Online simulated clinical practice
online-VMI: Online virtual medical interview
sEHR: Simulated electronic health records organized in Microsoft Excel
