# Supplementary material for: Alternative approaches for clinical clerkship during the COVID-19 pandemic: online simulated clinical practice for inpatients and outpatients—A mixed method
Source: BMC Med Educ. 2021 Mar 8;21:149. doi: 10.1186/s12909-021-02586-y (PMC7938264; doi:10.1186/s12909-021-02586-y)

**Supplementary Figures**

**Supplementary Figure 1.** **Daily flow of online clinical practice using sEHR.**

LMS: learning management system, sEHR: simulated electronic health records organized in Microsoft Excel

**
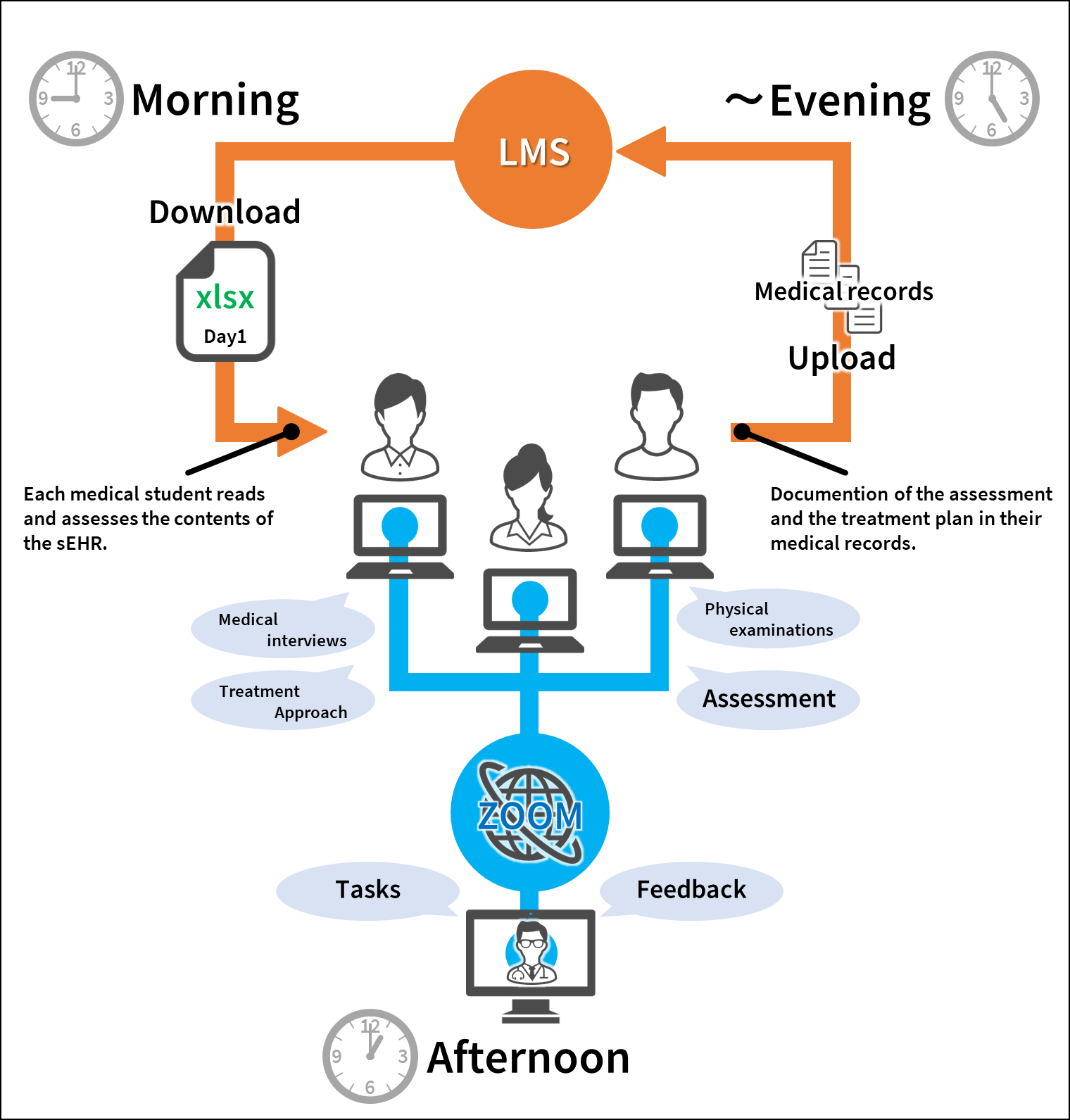
**

**Supplementary Figure 2. Electronic problem-based learning using the LMS.**

LMS: learning management system.

**
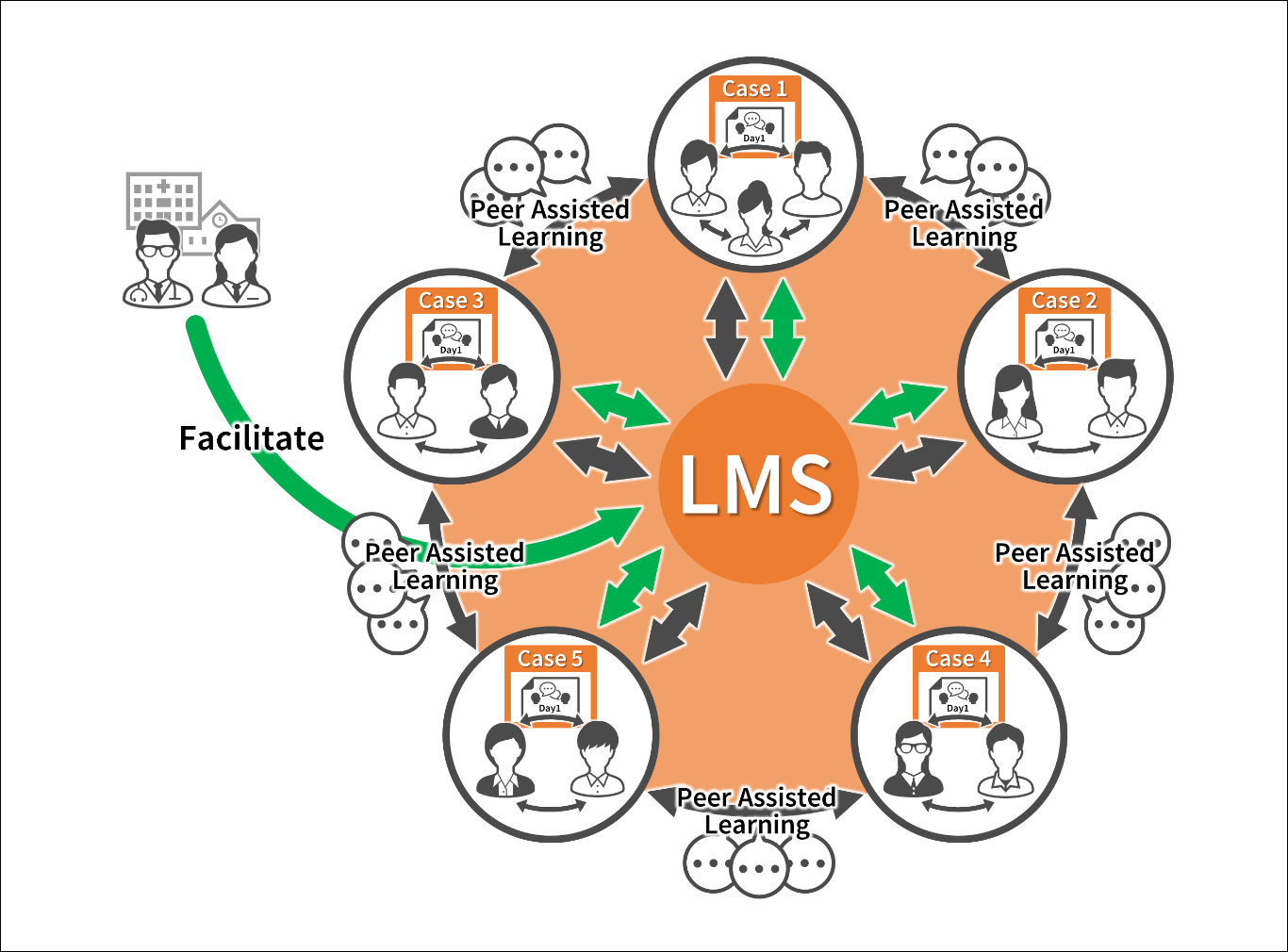
**

**Supplementary Figure 3. Process of online virtual medical interviews.**


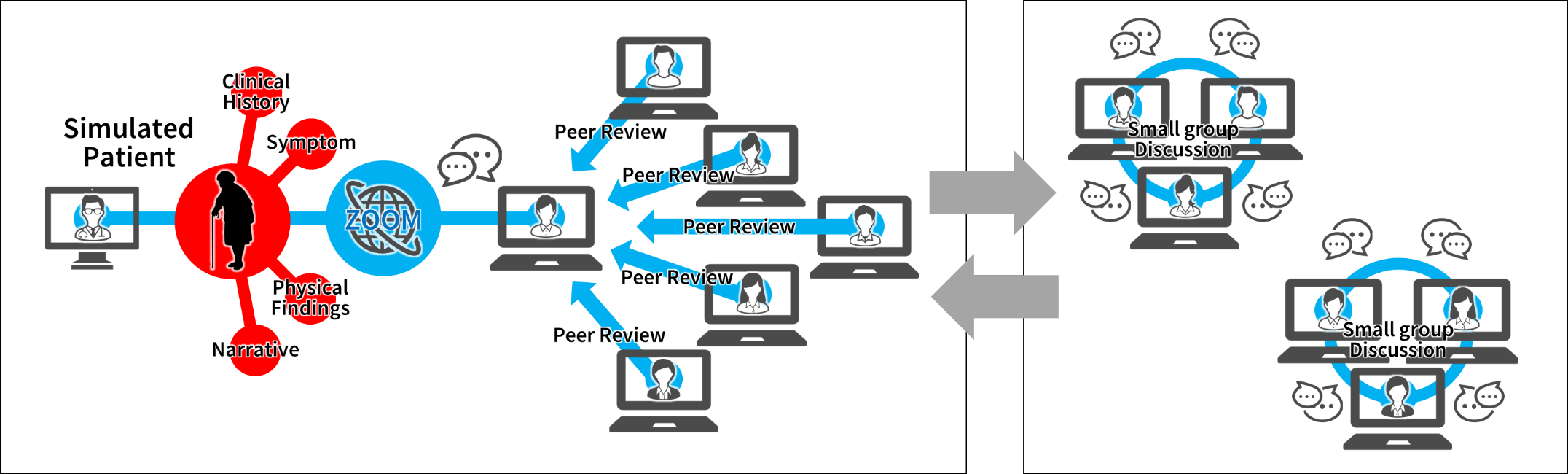


**Supplementary Figure 4.** **Flow diagram for the study.**

Online-sCP: online simulated clinical practice


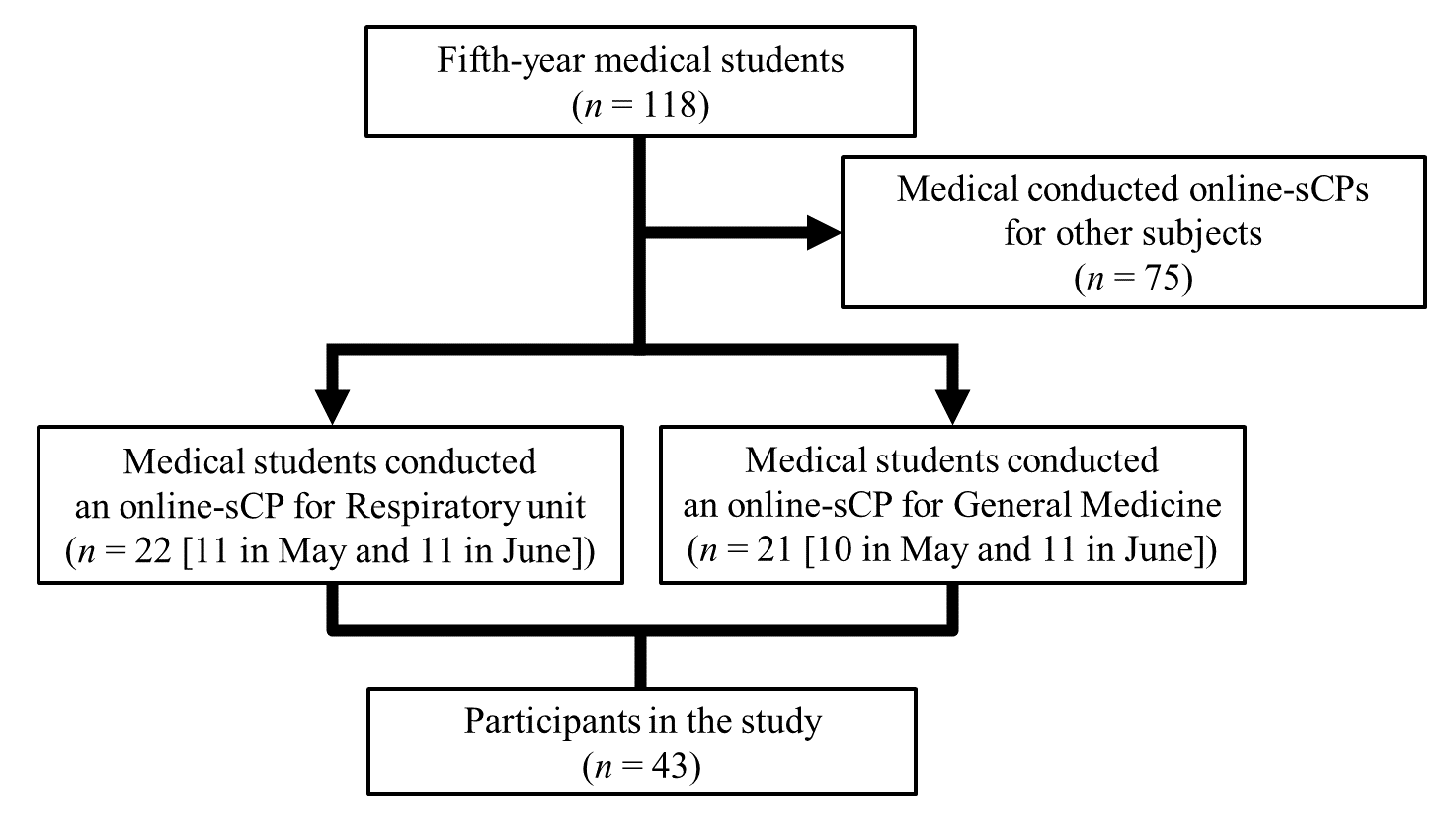

Supplement: Supplementary file 1 — Additional file 1 : Supplementary Fig. 1 Daily flow of online clinical practice using sEHR. LMS: learning management system, sEHR: simulated electronic health records organized in Microsoft Excel. Supplementary Fig. 2 Electronic problem-based learning using the LMS. LMS: learning management system. Supplementary Fig. 3 Process of online virtual medical interviews. Supplementary Fig. 4 Flow diagram for the study. Online-sCP: online simulated clinical practice. [file 12909_2021_2586_MOESM1_ESM.docx]
