# Supplementary material for: Alternative approaches for clinical clerkship during the COVID-19 pandemic: online simulated clinical practice for inpatients and outpatients—A mixed method
Source: BMC Med Educ. 2021 Mar 8;21:149. doi: 10.1186/s12909-021-02586-y (PMC7938264; doi:10.1186/s12909-021-02586-y)
Supplement: Supplementary file 2 — Additional file 2. [file 12909_2021_2586_MOESM2_ESM.docx]

February 9, 2021

Kai Philipp Schnabel

Editor

*BMC Medical Education*

Ref. No.: MEED-D-20-01208

**Title: Alternative approaches for clinical clerkship during the COVID-19 pandemic: Online clinical practice for inpatients and outpatients**—***A mixed method***

Dear Dr. Kai,

Thank you for your email dated February 4, 2021, regarding our manuscript, “**Alternative approaches for clinical clerkship during the COVID-19 pandemic: Online clinical practice for inpatients and outpatients**—***A mixed method.***”

We believe that our revised manuscript has suitably incorporated the reviewers’ suggestions and significantly improves on the previous version.

We trust that our paper is now suitable for publication in *BMC Medical Education*.

Thank you for considering our paper for publication. We look forward to hearing from you.

Sincerely,

Hajime Kasai, MD

Department of Respirology, Graduate School of Medicine

Chiba University, 1-8-1 Inohana, Chuou-ku Chiba 260-8670, Japan

Telephone: 81-43-222-7171 Ext.71014, Fax: 81-43-226-2176

E-mail: [daikasai6075@yahoo.co.jp](mailto:daikasai6075@yahoo.co.jp)

**Response to Editor’s comments**

**Comment:**

We apologize for the delay in operating your manuscript. While I am sure that your manuscript is about to be published and has improved a lot during your revision, I would kindly ask you to reconsider the two points made by Examiner 1. At least I would ask you to comment on them.

**Response:**

We are very happy to receive your email, and wish to express our strong appreciation for the reviewers’ comments on the previous revision, which have helped us to further improve our paper.

As requested, we have responded to each of Reviewers 1's comments below.

**Response to Reviewer #1’s comments**

**Comment:**

Dear Dr Kasai and colleagues,

Thank you for taking the time and effort to address our remarks and suggestions and documenting the changes so well. And sincere apologies for having taken so long with the review of the revised manuscript.

I find the revised manuscript a lot easier to read now and have no major remarks or objections. It is just two points that I would like to raise and submit for your consideration.

**Response:**

We wish to express our strong gratitude for your insightful comments.

**Comment #1:** In the very beginning of the (revised) discussion, you state this was the first study on online simulated clinical practice as alternative to face-to-face clerkships. I somewhat doubt this is true and believe that similar approaches have probably been there before the current pandemic even. Rather than making this claim, I suggest simply to re-state your study's goal and continue from there.

**Response:**

We thank you for this pertinent comment. We agree with your suggestion and have revised the text as follows

(p.17-18, 1st para. of Discussion):

**Original:** “To date, this is the first study that evaluates the effectiveness of online-sCP as an alternative to CC for outpatient and inpatient care. Two main findings have been observed.”

**Modified:** “This study evaluated the feasibility and effectiveness of online-sCP as an alternative to CC for outpatient and inpatient care, and identified the advantages and disadvantages of online-sCP for medical students. Its two main findings are as follows.”

**Comment #2:** Second, on page 23 in the last paragraph, you mention this study had two main limitations. If you asked me, I would say the main limitation is in the study design, relying on a single-site study with no control and based in parts on students' self-assessment. The self-study times from memory I find a relevant, but secondary factor. But that is only my take on this.

**Response:**

We thank you for raising this issue. As you pointed out, the biggest limitation is that our study design was constrained by the COVID-19 pandemic. Although this point was added to the Discussion in the previous revision, we now also include it as the first limitation.

(p.23-24, 6th para. of Discussion):

**Original:** “This study has two main limitations. First, since it was only conducted at a single university in Japan, the responses are subject to cultural bias and the participants’ learning environments. Second, the self-study time during CC and the period when CC was postponed were drawn from the students’ memories, because there were no subjective data, such as those kept in a portfolio. Students had been attending CCs for 2–3 months before the online-sCP, and their responses regarding their self-study time during CCs can be ambiguous. Students had been attending CCs for 2–3 months before the online-sCP, and their responses regarding their self-study time during CCs can be ambiguous.”

Modified: “This study has three main limitations. First, in terms of research design, this was a single-site study in an uncontrolled environment and relied partly on students’ self-assessment for data collection. Second, since it was only conducted at a single university in Japan, the responses are subject to cultural bias and the participants’ learning environments. Third, the self-study time during CC and the period when CC was postponed were drawn from the students’ memories, because there were no subjective data, such as those kept in a portfolio.”

Please note that the limitation concerning self-study time has already been addressed in response to Reviewer 2’s comment on the previous revision.

**Response to Reviewer #2’s comments**

**Comment:**

The manuscript has been appropriately revised according to my comments.

**Response:** We wish to express our strong appreciation for your comments on the previous revision, which helped us to improve our paper.
